# The interplay between polygenic score for tumor necrosis factor-α, brain structural connectivity, and processing speed in major depression

**DOI:** 10.1038/s41380-024-02577-7

**Published:** 2024-05-01

**Authors:** Kira Flinkenflügel, Marius Gruber, Susanne Meinert, Katharina Thiel, Alexandra Winter, Janik Goltermann, Paula Usemann, Katharina Brosch, Frederike Stein, Florian Thomas-Odenthal, Adrian Wroblewski, Julia-Katharina Pfarr, Friederike S. David, Eva C. Beins, Dominik Grotegerd, Tim Hahn, Elisabeth J. Leehr, Katharina Dohm, Jochen Bauer, Andreas J. Forstner, Markus M. Nöthen, Hamidreza Jamalabadi, Benjamin Straube, Nina Alexander, Andreas Jansen, Stephanie H. Witt, Marcella Rietschel, Igor Nenadić, Martijn P. van den Heuvel, Tilo Kircher, Jonathan Repple, Udo Dannlowski

**Affiliations:** 1 Institute for Translational Psychiatry, University of Münster, Münster, Germany; 2 Department of Psychiatry, Psychosomatic Medicine and Psychotherapy, University Hospital Frankfurt, Goethe University, Frankfurt, Germany; 3 Institute for Translational Neuroscience, University of Münster, Münster, Germany; 4 Department of Psychiatry and Psychotherapy, University of Marburg, Marburg, Germany; 5 Center for Mind, Brain and Behavior (CMBB), University of Marburg, Marburg, Germany; 6 Institute of Human Genetics, University of Bonn, School of Medicine & University Hospital Bonn, Bonn, Germany; 7 Department of Radiology, University of Münster, Münster, Germany; 8 Institute of Neuroscience and Medicine (INM-1), Research Center Jülich, Jülich, Germany; 9 Center for Human Genetics, University of Marburg, Marburg, Germany; 10 Core-Facility Brainimaging, Faculty of Medicine, University of Marburg, Marburg, Germany; 11 Department of Genetic Epidemiology in Psychiatry, Central Institute of Mental Health, Medical Faculty Mannheim, Heidelberg University, Heidelberg, Germany; 12 Connectome Lab, Department of Complex Trait Genetics, Center for Neurogenomics and Cognitive Research, Vrije Universiteit Amsterdam, Amsterdam Neuroscience, Amsterdam, The Netherlands; 13 Department of Child Psychiatry, Amsterdam University Medical Center, Amsterdam Neuroscience, Amsterdam, The Netherlands

**Keywords:** Neuroscience, Depression

## Abstract

Reduced processing speed is a core deficit in major depressive disorder (MDD) and has been linked to altered structural brain network connectivity. Ample evidence highlights the involvement of genetic-immunological processes in MDD and specific depressive symptoms. Here, we extended these findings by examining associations between polygenic scores for tumor necrosis factor-α blood levels (TNF-α PGS), structural brain connectivity, and processing speed in a large sample of MDD patients. Processing speed performance of *n* = 284 acutely depressed, *n* = 177 partially and *n* = 198 fully remitted patients, and *n* = 743 healthy controls (HC) was estimated based on five neuropsychological tests. Network-based statistic was used to identify a brain network associated with processing speed. We employed general linear models to examine the association between TNF-α PGS and processing speed. We investigated whether network connectivity mediates the association between TNF-α PGS and processing speed. We identified a structural network positively associated with processing speed in the whole sample. We observed a significant negative association between TNF-α PGS and processing speed in acutely depressed patients, whereas no association was found in remitted patients and HC. The mediation analysis revealed that brain connectivity partially mediated the association between TNF-α PGS and processing speed in acute MDD. The present study provides evidence that TNF-α PGS is associated with decreased processing speed exclusively in patients with acute depression. This association was partially mediated by structural brain connectivity. Using multimodal data, the current findings advance our understanding of cognitive dysfunction in MDD and highlight the involvement of genetic-immunological processes in its pathomechanisms.

## Introduction

Major depressive disorder (MDD) is considered one of the most prevalent and debilitating mental illnesses worldwide, with a lifetime prevalence of approximately 10.8% [1]. Impairments in processing speed (i.e., the ability to quickly acquire, process, and respond to information) are among the core features of acute MDD [2, 3] and have been associated with less favorable clinical trajectories [4] and poor treatment response [5]. Moreover, reduced processing speed is a major predictor of poor psychosocial functioning with adverse consequences for work, family, friends, and health [2, 4]. As most cognitive domains depend on rapid information transfer [6], dysfunctions in processing speed can lead to impairments in several other cognitive domains, such as attention, concentration, and memory [7, 8]. Some of these cognitive deficits persist even after remission [9] and may promote vulnerability to relapses [10]. Given all these findings, it is of particular clinical and scientific interest to understand the pathogenic mechanisms underlying reduced processing speed in MDD.

A promising way to gain more insights into these mechanisms is to study the neurobiological correlates of processing speed. Cognitive processes are thought to arise from a multitude of interacting brain regions rather than from individual brain regions alone [11, 12]. The human connectome, i.e., the network of all brain regions and their white matter (WM) connections, can be examined using diffusion-weighted imaging and network analyses [13]. Applying these methods, neuroimaging studies in healthy individuals have revealed robust associations between brain structural connectivity and cognitive performance, including processing speed [14–16], thus linking intact network connectivity to healthy cognitive functioning. Conversely, disruptions in network connectivity have been demonstrated in several mental disorders, including MDD [17–20], with deficits most pronounced in acutely depressed individuals [18]. These findings lead to the assumption that MDD-related alterations may also affect those brain networks associated with cognitive performance. Indeed, Gruber and colleagues [21] previously identified a structural subnetwork of frontotemporal fiber tracts that was positively related to processing speed. Within this network, the researchers further demonstrated associations between connectivity strength, processing speed, and depression severity, indicating that alterations in subnetwork-specific connectivity may provide a structural basis for impaired processing speed in MDD. However, despite recent advances in network neuroscience, little is known about the biological mechanisms *preceding* these brain structural alterations.

Brain structural connectivity, processing speed, and depression have been associated with dysregulations of the immune system [22–26], characterized by chronic production and expression of chemokines, acute phase proteins, and proinflammatory cytokines. Along with interleukin-1β and interleukin-6, tumor necrosis factor-α (TNF-α) is considered one of the key proinflammatory cytokines that has been linked to a number of somatic and mental diseases [27]. This is supported, for example, by studies reporting elevated TNF-α levels in peripheral blood and cerebrospinal fluid of MDD patients [28, 29], and anti-depressant effects of drug-induced TNF-α synthesis inhibition [30]. TNF-α interacts with the brain in a complex, bidirectional manner via cellular, neuronal, or endocrine pathways, where it can trigger neurotoxic processes, including inhibition of growth factors [31] or disruption of WM microstructure [32, 33]. While these immunological processes can be caused by exposure to environmental stressors (e.g., childhood maltreatment or negative life events) [34, 35], there is also evidence linking genetic variation to immunological dysregulation in MDD [36]. For example, twin studies have shown that the association between depression and increased immune activation can be attributed, at least in part, to shared genetic factors involved in the regulation of inflammatory processes [37, 38]. Furthermore, 34 of the 269 genes associated with depression in a genome-wide association study (GWAS) by Howard and colleagues [39] were found to be implicated in immunological processes, with some of them exhibiting cytokine-related features [40]. Likewise, there is evidence supporting a link between the polymorphism 308 (G/A) in the TNF-α gene and the risk of developing MDD [41, 42], as well as between polygenic scores for TNF-α blood levels and specific depressive symptoms [43]. So far, however, none of these studies have analyzed the associations with structural network connectivity and processing speed.

The main goal of the present study was to investigate the reciprocal relationships between genetic predisposition to TNF-α blood levels, brain structural connectivity, and processing speed in a well-powered sample of patients with MDD and HC. To this end, we first re-established previous findings of processing speed deficits in MDD patients [2, 3] and their association with structural brain connectivity [21]. We then extended these findings by evaluating the involvement of genetic-immunological processes in these associations using a polygenic score (PGS) for TNF-α blood levels. PGS estimate an individual’s genetic susceptibility to a certain trait or disease [44] and have been applied in various clinical and scientific contexts, including genome-wide gene-by-environment [45] and gene-by-gene interactions [46]. Based on the literature outlined above, we expect TNF-α PGS to be negatively associated with processing speed in MDD. In addition, we hypothesize that the association between TNF-α PGS and processing speed is mediated by connectivity strength in processing speed-related networks. Given that TNF-α PGS [43], structural connectome alterations [18, 21], and cognitive deficits [21, 47] have been particularly linked to *current* depressive symptomatology, we assume these associations to be most pronounced in acutely depressed patients.

## Materials and methods

### Participants

Our study consisted of *N* = 659 patients with MDD and *N* = 743 HC drawn from the ongoing Marburg-Münster Affective Disorders Cohort Study (MACS, (see [48] for the study protocol and [49] for the quality assurance protocol). Participants aged 18–65 years with West-European ancestry were recruited from January 09, 2015 to May 11, 2018 via newspaper advertisements or local psychiatric hospitals. MDD patients were included in our analyses if they were diagnosed with an acute (MDDa, *n* = 284) or a partially (MDDpr, *n* = 177) or fully remitted (MDDfr, *n* = 198) major depressive disorder. HC were included if they did not report a lifetime diagnosis of any psychiatric disorder. The Structured Clinical Interview for DSM-IV-TR [50] was used by trained interviewers to validate the diagnosis or lack thereof and to determine remission status (for more details see Supplement 1). See Supplement 2 for details on exclusion criteria and sample selection process and Supplement 3 for patients’ medication and comorbidities. Sample characteristics are provided in Table 1. Study procedures were approved by the Ethics Committees of the University of Münster (2014-422-b-S) and Marburg (07/14) following the Declaration of Helsinki. All participants signed informed consent before participation and received financial compensation.

**Table 1:** Demographic and clinical characteristics of the sample.

Variable	HC (*n* = 743)	MDDfr (*n* = 198)	MDDpr (*n* = 177)	MDDa (*n* = 284)	Statistic	*p*-value	Sig^a^
Sex (male: female)	261:482 (35:65%)	52:146 (26:74%)	69:108 (39:61%)	104:180 (37:63%)	8.18	0.042	−
Age	33.95 ± 12.54	36.11 ± 12.94	36.30 ± 12.90	36.30 ± 13.47	3.64	0.012	−
BMI	24.13 ± 4.40	25.10 ± 5.11	26.43 ± 5.80	26.23 ± 6.04	17.68	<0.001	B, C
TNF-α PGS	36.10 ± 0.07	36.09 ± 0.08	36.09 ± 0.08	36.10 ± 0.07	0.80	0.500	−
Processing speed	0.26 ± 0.92	-0.12 ± 0.94	−0.25 ± 0.91	-0.43 ± 1.09	41.93	<0.001	A, B, C, E, F
HDRS-21	1.41 ± 2.10	3.40 ± 3.45	7.93 ± 5.67	14.44 ± 6.29	759.28	<0.001	A, B, C, D, E, F
Hospitalizations	−	1.03 ± 1.41	1.69 ± 2.14	1.88 ± 2.11	11.62	<0.001	D, E
Comorbidities (yes: no)	−	67:131 (34:66%)	77:100 (43:57%)	146:138 (51:49%)	14.64	<0.001	E
Medication load	−	0.60 ± 0.98	1.50 ± 1.44	1.84 ± 1.50	50.54	<0.001	D, E, F

Except for sex and comorbidities, mean values and standard deviations are shown. Test statistics and *p* values were derived from general linear models (GLM) or Chi-square tests. Processing speed was calculated from five tests of a neuropsychological test battery using an exploratory principal component analysis.

*TNF-α* PGS Polygenic score for tumor necrosis factor-α, *BMI* Body mass index, *HDRS-21* Hamilton Depression Rating Scale 21 (total score), *HC* healthy controls, *MDDfr* patients with a fully remitted episode of major depressive disorder, *MDDpr* patients with a partially remitted episode of MDD, *MDDa* patients with an acute episode of MDD.

^a^Letters indicate significant (i.e., *p* < .05) differences in Bonferroni corrected post-hoc tests between HC and MDDfr (A), HC and MDDpr (B), HC and MDDa (C), MDDfr and MDDpr (D), MDDfr and MDDa (E), and MDDpr and MDDa (F).

### Assessment of processing speed and clinical characteristics

Processing speed was estimated based on the shared variance of five tests from a neuropsychological test battery that assess performance via 1. the time required to solve a certain task or 2. the number of items processed within a given amount of time: the Letter Number Sequencing Test (LNST), the Digit Symbol Substitution Test (DSST), the Trail Making Test (TMT-A & TMT-B), the Corsi Block Tapping Test (forward and backward), and the d2 Attention Test [21] (see Supplement 4).

To adjust for clinical features of MDD, we included the number of prior hospitalizations as a measure of cumulative illness severity and the presence of comorbid mental illness reported by patients in the SCID-I interview. In addition, the type and amount of current medication adherence were assessed using the Medication Load Index (MedIndex) [51]. The severity of current depressive symptomatology was measured in all participants using the Hamilton Depression Rating Scale [52].

### MRI data acquisition and preprocessing

MRI data were collected using 3 T whole-body MRI scanners at two scanning sites (Marburg: Tim Trio, Siemens, Erlangen, Germany; Münster: Prisma, Siemens, Erlangen, Germany). Due to a body coil exchange in Marburg, two dummy-coded variables (Marburg pre body-coil, Marburg post body-coil) with Münster as reference category were included as additional covariates in all analyses. See Supplement 5 for detailed information on acquisition parameters and Supplement 6 for preprocessing steps. Anatomical connectome reconstruction was performed using the CATO toolbox [53]. Hundred and fourteen nodes (i.e., cortical brain regions, based on the Cammoun subdivision of Freesurfer’s Desikan-Killiany atlas [54, 55]) were obtained from T1-weighted MRI, while edges (i.e., the mean fractional anisotropy (FA) of a fiber tract connecting a pair of nodes) were reconstructed from diffusion-weighted MRI. FA is an established parameter of WM integrity that has been associated with MDD and cognitive performance in previous neuroimaging studies [18, 56–58]. FA can range from 0 to 1, with higher values indicating direct diffusion and intact myelin [59]. Details on the reconstruction and quality assurance procedures are provided in Supplements 7 and 8.

### Genetic methods

Genotyping was performed in the MACS cohort with the Illumina Infinium PsychArray BeadChip, followed by quality control and imputation, as described earlier [60–62]. Briefly, quality control and population substructure analyses via multidimensional scaling (MDS) were conducted in PLINK v1.90 [63]. Genotype data was imputed to the 1000 Genomes phase 3 reference panel using SHAPEIT and IMPUTE2 [64–66]. Participants who were genetically related to other participants ($\hat{\pi }\ge 12.5$) in our sample were excluded from downstream analyses. PGS for TNF-α blood levels were computed using summary statistics from a recent GWAS by Ahola-Olli and colleagues [67]. The GWAS sample was independent of the MACS sample included in our study. For PGS calculation, single nucleotide polymorphism weights were estimated using PRS-CS with default parameters. The PRS-CS approach was chosen due to its advantages regarding prediction accuracy between the observed and predicted traits as compared to standard methods such as clumping and thresholding [68, 69]. The global shrinkage parameter set to φ = 1e^−6^ [68, 70]. The choice of the global shrinkage parameter was based on the assumption of a relatively low polygenicity of blood cytokine levels, including TNF-α, consistent with previous studies [43]. For analyses based on higher values for the shrinkage parameter, see Supplement 9.

### Statistical analyses

Demographic characteristics, clinical features, and factor structure of the neuropsychological tests were analyzed using IBM SPSS Statistics (version 28.0; IBM Corporation). Neuroimaging data were analyzed using the Network-based statistic toolbox (NBS [71]) implemented in Matlab 2019b [72]. All statistical models were corrected for sex and age. Models including neuroimaging data were further corrected for scanner settings. For analyses involving TNF-α PGS, body-mass-index (BMI) [67] as well as the first two ancestry components (C1, C2) extracted from the MDS analysis of genotype data based on their relative variance (Supplement 10), were added as covariates. If not otherwise stated, statistical tests were conducted at a two-sided significance level of α = 0.05.

Before testing our hypotheses, we employed a principle component analysis (PCA) to abstract from neuropsychological test scores to underlying cognitive domains. Given our focus on speed tests and based on our previous work [21], we expected a one-factor structure reflecting the underlying capacity of processing speed [6]. The number of factors to be extracted was determined according to the Kaiser-Guttman criterion, the Scree test, and parallel analysis [73]. Component scores were calculated using a linear regression approach. All subsequent analyses were performed with these component scores.

The first part of our analyses focuses on re-establishing processing speed deficits in MDD patients and their relation to structural brain connectivity across MDD and HC, as both results form the basis for our subsequent analyses on the role of TNF-α PGS. Therefore, we first calculated a general linear model (GLM) to examine whether the diagnostic groups (HC, MDDa, MDDpr, MDDfr) differed in their processing speed performance using the extracted component scores as dependent variable. Following our previous findings linking cognition to structural brain connectivity [21], we then used NBS to identify a network associated with processing speed in the whole sample, while accounting for diagnosis on top of the above-mentioned covariates. NBS estimates family-wise error (FWE)-corrected regression models to test the association between edge-wise connectivity strength, as measured by edge-wise mean FA, and the processing speed scores. The significance of an identified network was determined using a permutation test (5000 permutations, for more details, see Supplement 11). In case of significant results, mean FA across all edges from the identified network was extracted to SPSS.

In the second part of our analyses, we sought to investigate the association of TNF-α PGS with processing speed performance and structural brain connectivity. For this purpose, we again calculated a GLM, this time including diagnosis and TNF-α PGS as independent variables and processing speed as dependent variable. The main effect of TNF-α PGS as well as its interaction with diagnosis were analyzed. Finally, using a bootstrapping approach implemented in the SPSS macro PROCESS (http://www.processmacro.org), we tested our mediation hypothesis with TNF-α PGS as predictor variable (X), mean FA as mediator variable (M), and processing speed as outcome variable (Y). Direct as well as indirect effects were estimated. Significance is assumed if the 95% confidence interval (95% CI) does not include zero.

## Results

### Exploratory principal component analysis for processing speed

As expected, exploratory PCA yielded a single-factor structure that explained 51.05% of the variance of the cognitive tests, with factor loadings ranging from 0.62 to 0.79 (Supplement 12).

### Re-establishing processing speed deficits and their association with structural brain connectivity

The diagnostic groups differed significantly in the processing speed factor (*F*(3,1396) = 39.61, *p* < 0.001, partial *η*² = 0.078). Bonferroni-corrected post hoc tests revealed that patients with MDDa scored significantly worse than MDDfr (MD = −0.30, *p* = 0.001) and HC (MD = −0.61, *p* < 0.001). HC scored significantly higher compared with MDDpr (MD = 0.43, *p* < 0.001) and MDDfr (MD = 0.31, *p* < 0.001). MDDfr and MDDpr as well as MDDa and MDDpr did not differ from each other (all *ps* $\ge$ 0.185).

**Fig. 1:**
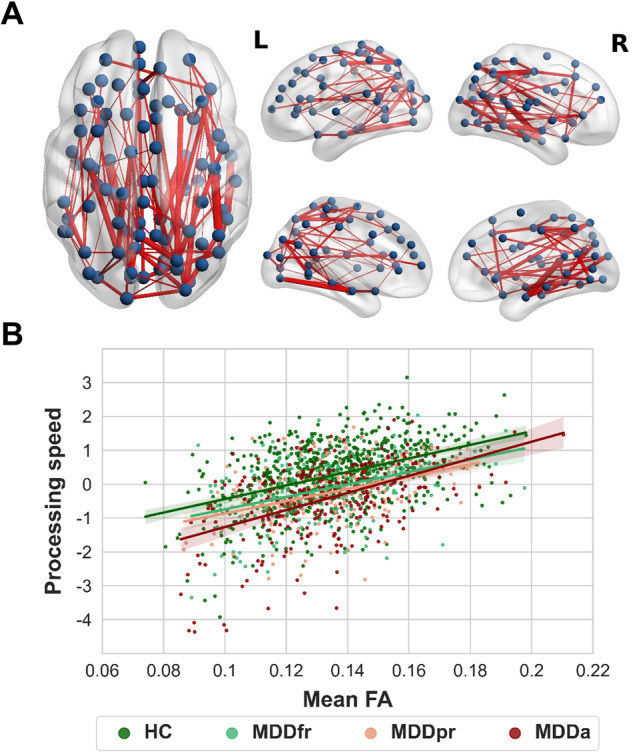
Network of white matter fiber tracts that are positively associated with processing speed across diagnostic groups. **A** Dorsal, lateral, and medial views of the network of white matter fiber tracts in which connectivity strength, measured with edge-wise fractional anisotropy (FA), was positively associated with processing speed (z-score) across diagnostic groups. The network was identified using the Network-based statistic (NBS) toolbox (NBS *t*-threshold = 2.0, *p*_FWE_ < 0.001), while correcting for age, sex, and scanner site. See Supplement 13 for details on participating anatomical brain regions. Network plots were created using BrainNet Viewer. **B** Scatterplot depicting the positive association between mean FA and processing speed across patients with acute (MDDa), partially (MDDpr), and fully remitted (MDDfr) major depressive disorder and healthy controls (HC). A more positive processing speed score represents higher processing speed performance.

The NBS analysis conducted in the whole sample identified a network of edges whose FA was significantly associated with processing speed (NBS *F*-threshold = 4.0, 206 edges, 106 nodes, *p*_FWE_ < 0.001, partial *η*^2^ = 0.052). Post-hoc analyses revealed a positive association between processing speed and connectivity strength (i.e., edge-wise FA) (NBS *t*-threshold=2.0, 121 edges, 89 nodes, *p*_FWE_ < 0.001, partial *η²*=0.163, Fig. 1). The network comprised a large proportion of fronto-parietal edges (22%) (Supplement 13). See Supplement 14 for analyses based on different NBS *t*-thresholds.

### Association between processing speed and TNF-α PGS

**Fig. 2:**
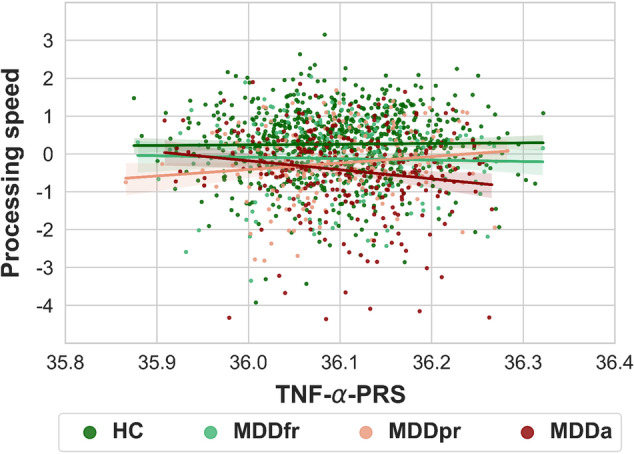
Interaction effect between TNF-α PGS and diagnosis on processing speed. **Note**. The figure depicts the significant interaction effect between polygenic score for tumor necrosis factor-α (TNF-α PGS) and diagnosis on processing speed. Patients with acute major depressive disorder (MDDa) showed a negative association between TNF-α PGS and processing speed, whereas no association was found in patients with a partially (MDDpr) or fully remitted (MDDfr) depressive episode and healthy controls (HC). Processing speed (z-score) was calculated from five tests of a neuropsychological test battery using exploratory principal component analysis. A more positive processing speed score represents higher processing speed performance.

The GLM revealed no main effect of TNF-α PGS (*p* = 0.599). However, we observed a significant TNF-α PGS × diagnosis interaction effect (*F*(3,1389) = 4.40, *p* = 0.004, partial *η*² = 0.009), which was driven by a negative association between TNF-α PGS and processing speed in the MDDa group (*B* = −2.41, *p* = .008, partial *η*² = 0.034; Bonferroni-corrected), whereas no association was found in the MDDpr (*p* = 0.936), MDDfr (*p* > 0.999), and HC group (*p* > 0.999) (Fig. 2).

### Structural brain connectivity as a mediator of the association between TNF-α PGS and processing speed

**Fig. 3:**
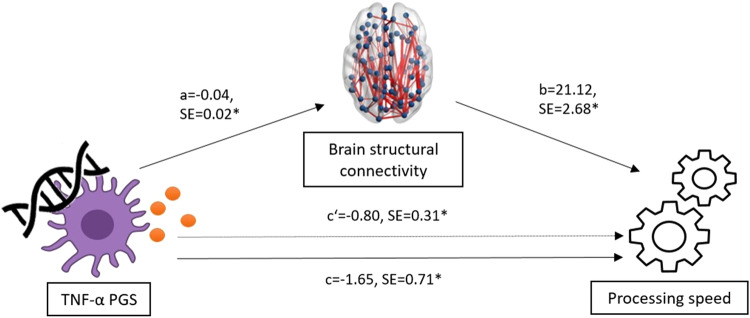
Brain structural connectivity mediates the association between polygenic score for tumor necrosis factor-α (TNF-α PGS) and processing speed in acutely depressed patients. The figure depicts the mediation model with TNF-α PGS as a predictor variable, structural brain connectivity (measured by mean fractional anisotropy) as a mediator variable, and processing speed as an outcome variable in acutely depressed patients. A higher processing speed score represents higher processing speed performance. Unstandardized coefficients and standard errors for each path of the mediation model are presented. Note that c represents the direct effect and c‘ the indirect effect. * indicates significance at *p* < 0.05.

As we found a significant association between TNF-α PGS and processing speed exclusively in patients suffering from acute depression, the mediation model was tested only in the MDDa subgroup. The analysis showed a significant negative association between TNF-α PGS and processing speed (coeff = −2.45, 95%-CI [−3.97, −0.93], SE = 0.77, *t* = −3.17, *p* = 0.002), as well as between TNF-α PGS and mean FA (coeff = −0.04, 95% CI [−0.07, −0.01], SE = 0.02, *t* = −2.52, *p* = 0.012). Furthermore, we observed a significant negative indirect (mediated) effect of TNF-α PGS on processing speed through mean FA (coeff = −0.80, 95% CI [−1.45, −0.25], SE = 0.31). Lastly, the model yielded a significant direct effect of TNF-α PGS on processing speed (coeff = −1.65, 95% CI [−3.05, −0.24], SE = 0.71), indicating that the association between TNF-α PGS and processing speed was only partially mediated by mean FA (Fig. 3).

### Robustness checks and exploratory analyses

Correction for influential data points, clinical characteristics within patients, and additional MDS components yielded a comparable pattern of results (Supplement 15). Furthermore, effects did not differ by sex (Supplement 16). Leave-one-site-out cross validation revealed good generalizability and prediction accuracy of the mediation model to unknown data (Supplement 17).

## Discussion

To our knowledge, this is the first study to analyze the reciprocal associations between PGS for TNF-α blood levels, brain structural network connectivity, and processing speed in a well-powered sample of MDD patients and HC. Employing state-of-the-art network analyses, we re-established our previous findings on the human connectome, showing a positive association between brain network connectivity strength and processing speed in patients with MDD and HC [21]. The present study extends these findings by providing evidence for a genetic-immunological mechanism that may underlie these structural links. More specifically, we demonstrated a negative association between TNF-α PGS and processing speed in patients suffering from acute MDD, while no association was found in partially or fully remitted patients and HC. Of note, this association was mediated, at least partially, by structural brain connectivity. Although effect sizes were small to moderate, they are consistent with effect sizes found in previous neuroimaging [74] and genetic studies [75]. Our results appear to be robust, as they remained unchanged when clinical characteristics in the MDD sample were taken into account. Overall, the current data suggest genetic-immunologic mechanisms to play a role in impaired processing speed in patients with acute depression.

Corroborating previous findings [2, 3], we demonstrated slowed processing speed in patients with MDD compared to HC. The deficits were most pronounced in patients with acute MDD but still detectable in partially or fully remitted patients, underscoring the persistence of cognitive dysfunction in depression [9]. According to established cognitive theories, processing speed serves as a basic mental capacity that affects performance - and thus deficits - in several other cognitive domains, such as attention, concentration, and memory [7, 8]. Given the detrimental impact of persistent cognitive dysfunction on disease outcomes [4] and psychosocial functioning [2, 4], its effective and targeted treatment in the context of antidepressant therapy is of high clinical relevance.

On a neuronal level, cognitive dysfunction in MDD has been associated with alterations in the structural connectome [21], that is, the network of all brain regions and their WM connections [13]. In line with this evidence, we identified a structural network, whose WM fiber connections were positively associated with processing speed in patients with MDD and HC. The network comprised a large proportion of fronto-parietal fibers and hubs (i.e., brain regions with the highest degree), such as the inferior parietal cortex and parahippocampal gyrus, consistent with the involvement of these brain areas in cognitive processes [76, 77]. Along with previous findings [18, 56], we propose that reduced FA, as an indicator of decreased information exchange between these brain areas and impaired network architecture, might provide a structural basis for processing speed deficits in MDD and HC.

While previous network neuroscience has mainly focused on the mere association between cognitive (dys-)function and structural brain alterations, the present study adds a possible biological mechanism for those alterations. More specifically, we demonstrated a triad of genetic predisposition to TNF-α blood levels, structural brain connectivity, and processing speed in patients with acute MDD. TNF-α is a proinflammatory cytokine that plays a critical role in the development and maintenance of depressive symptoms, including cognitive dysfunction [28, 31]. According to established theories [78], chronically elevated levels of proinflammatory cytokines, such as TNF-α, could lead to prolonged hypothalamic–pituitary–adrenal (HPA) axis activation via stimulating cortisol release and counteracting its negative feedback functions. In fact, HPA axis dysfunction, including hypercortisolemia, is a common feature of MDD that has been linked to its pathophysiology [79, 80], including cognitive dysfunction [79]. Cortisol and other glucocorticoids have been found to alter oligodendrocyte function by inhibiting their differentiation and myelogenesis [81]. These neurotoxic processes might affect WM microstructure [82, 83], leading to alterations in the structural connectome [84]. However, given the complexity of the (bidirectional) relationship between cytokines and HPA-axis [85], the cross-sectional nature of the current study, and the lack of state-dependent immunological and endocrinological markers, this conclusion remains speculative. Future longitudinal studies should extend our findings by adding biological serum markers in repeated-measures designs.

Nevertheless, the genetic perspective allows us to draw tentative conclusions about the causal directions of the effects observed since we can assume that, during periods of acute depression, TNF-α PGS leads to changes in the brain and in processing speed and not vice versa. Indeed, MDD is a multifactorial disorder that arises and is maintained by a complex interaction between environmental and genetic factors [86]. Increasing evidence from genetic research suggests that certain cytokine polymorphisms, including TNF-α, may be involved in the pathogenesis of MDD and related deficits [40, 43, 87]. Consistent with this assumption, we showed that TNF-α PGS is associated with impaired processing speed and that this association is partially mediated by structural brain connectivity. Notably, the effects appeared to be exclusive to patients suffering from acute MDD, as no associations have been found in (partially) remitted patients and HC. This might be explained by the fact that acute patients showed a greater variance in their processing speed performance compared with the other diagnostic groups. Similarly, this finding could indicate that gene × environment interactions are more likely to result in the observed cognitive deficits rather than genetic mechanisms alone. Future studies should consider additional measures (e.g., questionnaires, serum cortisol) in their analyses to evaluate the contribution of environmental factors, especially stress-related factors, to this association.

While our analyses focus on TNF-α as a key proinflammatory cytokine linked to the development and persistence of MDD [27, 88], we also recognize the involvement of other inflammatory markers in depressive symptoms, including cognitive impairment. For instance, previous studies have demonstrated associations between several proinflammatory interleukins such as IL-6 and IL-1β and reduced processing speed performance [89] and structural brain alterations [25], making generalizations of our results to other inflammatory markers likely. On the other hand, evidence also suggests the specificity of inflammatory markers in depressive symptoms by indicating that these associations are not universally applicable across all cytokines [43, 90]. Therefore, future studies are warranted to clarify the specificity of cytokines and their genetic predispositions implicated in the complex interplay between structural brain connectivity and processing speed performance in acute MDD.

The main strengths of the current study are the large and well-characterized sample and the integration of multimodal, i.e., genetic, cognitive, and magnetic imaging-derived brain network data. Nonetheless, some limitations should be noted. As mentioned above, our conclusions must be interpreted in light of the lack of state-dependent immunologic measures and the cross-sectional nature. In particular, mediation models imply causal relationships between variables. As we analyzed both cognitive and imaging data cross-sectionally, we cannot exclude the possibility of reverse causal relationships or the influence of omitted variables [91]. Another limitation arises from the operationalization of processing speed, as the included neuropsychological tests might also tap into other cognitive domains [92]. Regarding genetics, it must be acknowledged that the calculation of PGS was based on a relatively small GWAS consisting of Finns [67]. Since Finns have a Siberian ancestry [93], this may have led to a divergence from the Western-European ancestry of our MACS sample [43]. Further, our interpretations rely on the assumption of low polygenicity of TNF-α blood levels. While the interaction effect between diagnosis and TNF-α PGS on processing speed became significant for two of the three tested shrinkage parameters, mediation analysis yielded less consistent results. This might limit the generalizability of our findings. Finally, we did not directly measure TNF-α blood levels, nor did our PRS consider environmental factors, which also play an important role in the multifactorial nature of inflammation, depression, and cognitive performance [23, 94]. For instance, in our previous work, we showed that childhood maltreatment - one of the most important environmental risk factors for depression - and polygenic risk for MDD were independently associated with cognitive dysfunction [95]. Although we acknowledge these limitations, our approach does shed new light on a possible genetic pathway contributing to the link between structural brain connectivity and processing speed. This, in turn, might represent a starting point for future studies that should replicate our findings and extend their analyses to include serum markers of proinflammatory cytokines.

In conclusion, the current study provides evidence of a shared link between genetic predisposition to TNF-α blood levels, altered brain structural connectivity, and processing speed deficits in patients suffering from acute depression. Based on genetic-immunological, cognitive, and magnetic imaging-derived brain network data, we demonstrated that TNF-α PGS is associated with reduced processing speed in patients with acute MDD and that this association is partially mediated by brain structural connectivity. The current findings advance our understanding of cognitive dysfunction in MDD and could provide novel targets for the development of therapeutic interventions. Longitudinal studies are required to further unravel the genetic, immunological, environmental, and neural mechanisms of cognitive deficits and their interaction in MDD.

## Supplementary information

Supplemental material

## Data Availability

The datasets generated and/or analyzed during the current study are available from the corresponding author on reasonable request.

## Code availability

Analyses were performed in Matlab 2019b. Codes will be available upon request and communication with the corresponding author.
