# Supplementary material for: The interplay between polygenic score for tumor necrosis factor-α, brain structural connectivity, and processing speed in major depression
Source: Mol Psychiatry. 2024 May 1;29(10):3151–9. doi: 10.1038/s41380-024-02577-7 (PMC11449800; doi:10.1038/s41380-024-02577-7)
Supplement: Supplementary file 1 — Supplemental material [file 41380_2024_2577_MOESM1_ESM.docx]

**Supplements**

**Supplement 1: Definition of remission status**

Patients' remission status was determined according to the DSM-IV criteria [1]. A current depressive episode requires the presence of ≥ 5 symptoms that occur almost every day for two weeks or more during the last four weeks and represent a significant impairment in the patients' daily life. At least one of these symptoms is either a depressed mood or loss of interest and pleasure. Further symptoms include alterations in sleep (insomnia or hypersomnia) and appetite, psychomotor agitation or retardation, fatigue or loss of energy, feelings of worthlessness or guilt, difficulty concentrating or making decisions, and recurrent thoughts of death or suicide. Partial remission was defined as either 1) the persistence of some symptoms after a depressive episode, but no longer fully meeting DSM-IV criteria, or 2) the absence of depressive symptoms, with a period of remission shorter than two months. Full remission was defined as the absence of depressive symptoms for at least two months.

**Supplement 2: Choosing participants from the Marburg-Münster Affective Disorders Cohort Study (MACS)**

Our sample was drawn from the third data freeze (15.11.2021) of the MACS, which consisted of *N*=2254 participants aged 18 to 65 years with Western-European ancestry. Exclusion criteria included any history of neurological or medical conditions, a lifetime diagnosis of schizophrenia, schizoaffective, bipolar, or substance disorder, and general MRI contraindications. *N*=381 participants had to be removed from the data as they suffered from a diagnosis that was not relevant to the present study. Of the *n*=1873 participants, *n*=899 had a diagnosis of MDD and *n*=974 were HC. Due to missing MRI images or insufficient data quality, *n*=148 further participants had to be excluded from the sample (MDD *n=*804, HC *n*=921). An additional *n*=276 participants were removed because of clinical drop-out criteria or missing neuropsychological, genetic, or other important data (MDD *n=*675, HC *n*=774). Finally, *n*=47 further participants had to be excluded because they were genetically related to other participants. This resulted in the final sample of MDD *n*=659 and HC *n*=743.

**Supplement 3: Patients’ medication and comorbidities**

| **Table S3.T1.** *Prescribed psychiatric medications in MDD patients.* | | | |
| --- | --- | --- | --- |
| **Medication** | **MDDfr (*n*=189)** | **MDDpr (*n*=177)** | **MDDa (*n*=284)** |
| SNRI | 29 | 46 | 95 |
| SSRI | 22 | 47 | 89 |
| NDRI | 2 | 5 | 13 |
| NaSSA | 5 | 21 | 34 |
| NaRI | 0 | 0 | 1 |
| TCA | 2 | 9 | 23 |
| MAOI | 1 | 3 | 0 |
| Agomelatine | 3 | 11 | 16 |
| Lithium | 3 | 3 | 8 |
| Neuroleptic | 10 | 38 | 74 |
| **Note.** Numbers represent the absolute number of patients; MDDfr=patients with a fully remitted episode of major depressive disorder, MDDpr=patients with a partially remitted episode of MDD, MDDa=patients with an acute episode of MDD, SNRI=selective serotine-norepinephrine reuptake inhibitor, SSRI=selective serotonin reuptake inhibitor; NDRI=norepinephrine-dopamine reuptake inhibitor, NaSSA=noradrenergic and specific serotonergic antidepressant, NaRI=noradrenaline reuptake inhibitor, TCA=tricyclic antidepressants, MAOI=monoamine oxidase inhibitors. | | | |

| **Table S3.T2.** *Details on patients’ comorbidities.* | | | |
| --- | --- | --- | --- |
| **Diagnosis** | **MDDfr (*n*=189)** | **MDDpr (*n*=177)** | **MDDa (*n*=284)** |
| Anxiety disorder | 60 | 84 | 145 |
| Dysthymia | 2 | 7 | 19 |
| Eating disorder | 10 | 11 | 28 |
| Impulse-Control Disorders | 0 | 0 | 1 |
| Brief psychotic disorder | 1 | 0 | 1 |
| Delusional disorder | 1 | 1 | 0 |
| Somatoform disorders | 3 | 7 | 8 |
| Substance-related disorder | 13 | 3 | 15 |
| **Note.** Numbers represent the absolute number of patients; MDDfr=patients with a fully remitted episode of major depressive disorder, MDDpr=patients with a partially remitted episode of MDD, MDDa=patients with an acute episode of MDD | | | |

**Supplement 4: Details on neuropsychological tests**

Processing speed was estimated based on the shared variance of five tests from the MACS neuropsychological test battery that assess performance via 1) the time required to solve a certain task or 2) the number of items processed within a given amount of time [2].

Digit Symbol Substitution Test (DSST)

The Digit Symbol Substitution Test [3] is a subtest of the Wechsler Adult Intelligence Scale – Revised [4] and is considered a measure of processing speed. The participant is asked to match a series of single-digit numbers with fixed abstract symbols within 90 seconds. The resulting test score is based on the correct number of number-symbol substitutions.

Trail Making Test (TMT-A & TMT-B)

The Trail Making Test [5] consists of two parts. In the first part of the test (TMT-A), the participant is asked to connect randomly arranged numbers (1 - 25) in ascending order as quickly as possible. This score is considered a measure of processing speed and visual scanning. In the second part (TMT-B), the participant needs to alternate between numbers and letters both in sequential order (e.g., 1 - A - 2 - B - ... - 13). The time in seconds required by the participant is used as the score for each test.

Corsi Block Tapping Test (forward and backward)

The Corsi Block Tapping Test [6] is supposed to measure visual-spatial short-term and working memory. The participants are presented with a board on which nine blocks are arranged irregularly. The experimenter taps the blocks in a specific order and the participant is immediately asked to tap the identical blocks in the same order (Corsi Block Tapping Test forward). In the Corsi Block Tapping Test backward test, the participant needs to tap the blocks in exactly the opposite order. Throughout each task, the sequences become increasingly longer. The two test scores are calculated from the number of correctly remembered items.

d2 Attention Test

The d2 Attention Test [7] measures selective attention and concentration ability. The participants have to go through 14 lines consisting of 47 letters ("d" or "p") and mark the d's that have exactly 2 dashes. For each line, the participant has 20 seconds. Concentration performance is calculated on the sum of the marked letters minus the letters that were omitted or incorrect.

Letter Number Sequencing Test (LNST)

The Letter Number Sequencing Test is again a subtest of the Wechsler Adult Intelligence Scale – Revised [4] and is considered a measure of verbal working memory capacity. The test consists of four different rounds in which the participant has to sort a verbally given sequence of numbers and letters in a different order. First, the participant is asked to sort the sequence by naming all the numbers in ascending order and then all the letters in alphabetical order. The sequences of letters and numbers get longer with each round. The result is defined by the number of correct answers.

**Supplement 5: MRI data acquisition**

The structural T1 and diffusion-weighted (DW) images were obtained using 3T whole-body MRI scanners (Marburg: Tim Trio, 12-channel head matrix Rx-coil, Siemens, Erlangen, Germany; Münster: Prisma, 20-channel head matrix Rx-coil, Siemens, Erlangen, Germany) with a GRAPPA acceleration factor of two. T1-data was acquired from high-resolution, T_1_-weighted three-dimensional fast gradient echo sequences (MPRAGE) (repetition time (TR)=2130ms, echo time (TE)=2.28ms, inversion time (TI)=900ms, flip angle=8°) with an isotropic voxel size of 1×1×1mm³. For DW images, fifty-six axial slices, 2.5-mm thick with no gap, were obtained with an isotropic voxel size of 2.5×2.5×2.5mm³ (TE=90ms, TR=7300ms). Five non-DW images (*b_0_*=0) and 2×30 DW images with a *b*-value of 1000 s/mm² were measured.

**Supplement 6: Preprocessing of diffusion-weighted images**

Preprocessing of diffusion tensor imaging data was carried out in FSL6.0.1 (http://fsl.fmrib.ox.ac.uk/fsl/fslwiki/, FMRIB, Oxford Center for Functional MRI of the Brain, University of Oxford, Department of Clinical Neurology, John Radcliffe Hospital, Oxford, United Kingdom) [8–10]. Diffusion-weighted images (DWI) were realigned and corrected for head motion and eddy artifacts. Diffusion tensor imaging models the measured signal from a voxel by a single tensor that describes the diffusion signal as a preferred diffusion direction per voxel. The CATO toolbox [11] (applied to reconstruct the anatomical connectome (see Supplement 6)), uses the informed RESTORE algorithm [12, 13], which estimates the tensor while identifying and eliminating outliers during fitting. This reduces the influence of physiological noise artifacts on the DTI modeling. Based on the diffusion profiles, WM tracts were reconstructed using deterministic tractography. For this purpose, eight seeds per voxel were started, and a tractography streamline was constructed for each seed following the main diffusion direction from voxel to voxel. Stopping criteria included 1) reaching a voxel with fractional anisotropy (FA) <0.1, 2) reaching a sharp curve of >45°, 3) reaching a gray matter voxel, or 4) leaving the brain mask [14]).

**Supplement 7: Anatomical connectome reconstruction**

Anatomical connectome was reconstructed as described in our previous work [15, 16], using the CATO toolbox [11]. We obtained an anatomical brain network including 114 regions based on the Desikan-Killiany Atlas of FreeSurfer [17–19] and WM streamlines connecting these regions for each participant. In line with previous studies and due to the low DWI signal-to-noise ratio in subcortical brain areas [16, 20], we chose a subdivision of this atlas containing only cortical regions. For WM streamlines reconstruction, we used a deterministic streamline tractography based on the Fiber Assignment by Continuous Tracking (FACT) algorithm [21] that has shown to provide an adequate balance between false-negative and false-positive fiber reconstructions [22]. Connections between two nodes (i.e., brain regions), were considered if they were connected by at least three tractography streamlines to balance the sensitivity and specificity of the resulting connectivity matrices [23, 24]. We then stored network information for each participant in a structural connectivity matrix. The rows and columns of the connectivity matrix represent nodes, while the matrix entries represent edges that were weighted with FA.

**Supplement 8: Quality control procedure for connectivity matrices**

Following the approach of [25], several criteria were used to identify outliers within the connectivity matrices to ensure sufficient quality of the matrices. The criteria for detecting outliers comprised 1) the average number of streamlines, 2) the average FA, 3) the average prevalence of each participant's connections (low value if the participant has "odd" connections), and 4) the average prevalence of each participant's connected brain regions (high value if the participant misses frequent connections). Quartiles (Q1, Q2, Q3) and the interquartile range (IQR=Q3-Q1) were computed for each metric. A data point was considered an outlier if its value for any of the four metrics was below Q1-1.5*IQR or above Q3+1.5*IQR.

**Supplement 9: Results using different polygenic shrinkage parameters for TNF-α PGS**

| **Table S9.T1.** *Comparison of different polygenic shrinkage parameters for TNF-α PGS.* | | | |
| --- | --- | --- | --- |
| **Diagnosis** | **φ=1e^-2^** | **φ=1e^-4^** | **φ=1e^-6^** |
| **Interaction effect of TNF-α PGS and diagnosis on processing speed** | | | |
| *F* value | 1.89 | 2.86 | 4.40 |
| *p* value | <.130 | .036 | .004 |
| partial *η²* | 0.004 | 0.006 | 0.009 |
| **Mediation analysis: Total effect of TNF-α PGS** | | | |
| coeff | -0.04 | -2.94 | -2.45 |
| 95%-CI | [-0.11, 0.02] | [-0.56, -0.03] | [-3.97, -0.93] |
| **Mediation analysis: Direct effect of TNF-α PGS** | | | |
| coeff | -0.04 | -0.23 | -1.65 |
| 95%-CI | [-010, 0.02] | [-0.48, 0.02] | [-3.05, -0.24] |
| **Mediation analysis: Indirect effect of TNF-α PGS** | | | |
| coeff | -0.01 | -0.06 | -0.80 |
| 95%-CI | [-0.03, 0.02] | [-1.76, 0.04] | [-1.45, -0.25] |
| **Note.** The table shows results from analyses using different polygenic shrinkage parameters φ for polygenic score for tumor necrosis factor-α (TNF-α PGS). The association with processing speed was significant for two of the three strictest shrinkage parameters, whereas the use of a liberal threshold did not provide significant results. The indirect and direct effects of the mediation analysis provided significant results only for the strictest threshold. The total effect became significant for the strictest and second strictest thresholds. 95%-CI=95% confidence interval. | | | |

**Supplement 10: MDS component analysis**

**Figure S10.F1.** *Scree plot depicting the relative variance of MDS components resulting from the genetic population stratification analyses*


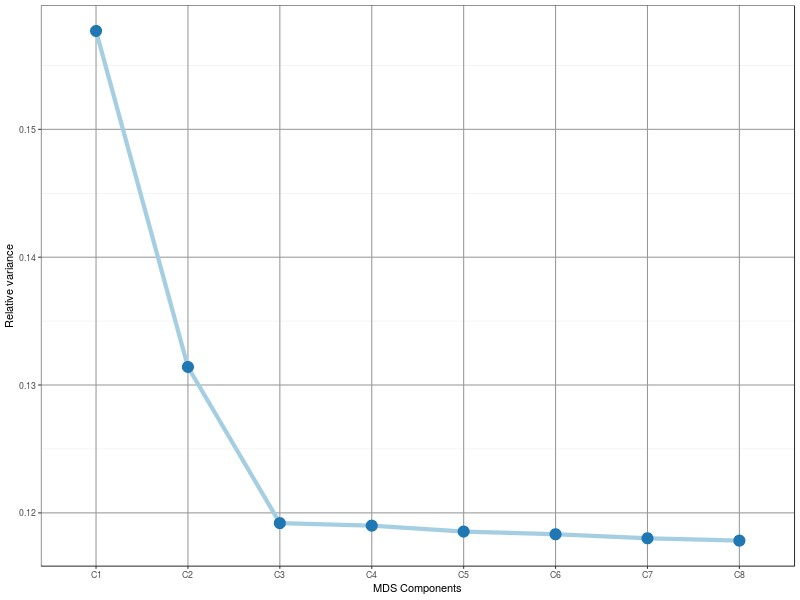


**Note.** The figure shows the number of multidimensional scaling (MDS) components as a function of their relative variance. All MDS components whose relative variance lie in front of the curve of the line in the Scree test were considered relevant. The Scree test suggests the extraction of two MDS components.

**Supplement 11: Details on the network-based-statistics (NBS) method**

We employed NBS [26] to identify networks of edges related to cognitive performance. NBS identifies a cluster-level effect by performing univariate mass tests at the edge level, controlling for FWE. The analysis proceeded as follows: Each edge was first assigned a *F*-value reflecting the association between mean FA of that edge and processing speed. The *F*-values were thresholded at *F=*4.0 to select all suprathreshold edges. The largest network of suprathreshold edges was then selected to identify the most robust network of edges associated with the processing speed factor. This procedure was then repeated 5000 times in permutation tests to identify an empirical null distribution describing the size of networks [27]. The resulting *p-*value indicates the proportion of permutations in which the largest network in the null distribution was larger than the one initially identified. The analysis was repeated with a thresholded at *t*=2.0 to specify the direction of the effect.

**Supplement 12: Details on principal component analysis (PCA)**

The PCA was performed according to the recommendations in [28]. Our procedure can be divided into four steps: 1) data preparation, 2) assessment of the adequacy of conducting PCA, 3) determining the number of factors to extract, and 4) extraction of the factor scores.

## Data preparation

We included seven scores from four different neuropsychological tests (see Supplement 3) in the PCA. Given our focus on speed tests and based on our previous work [15] , we expected a one-factor structure reflecting the underlying capacity of processing speed [2]. Scores from TMT-A and TMT-B were inverted by multiplying them with -1 to ensure that higher values indicate higher performance in all tests. All variables were z-standardized before being entered into the analysis.

Assessment of adequacy

To assess the adequacy of conducting exploratory factor analysis, we performed Bartlett's test of sphericity and the Kaiser-Meyer-Olkin (KMO) test. Bartlett's test of sphericity tests whether the observed variables are correlated with each other by comparing the observed correlation matrix with an identity matrix. The test was significant (*χ^2^*=3375.717, *p*<.001), suggesting intercorrelation between the variables. The KMO test determines the suitability of the data for factor analysis by estimating the proportion of common variance between all observed variables. KMO values range from 0 to 1, with values below 0.6 considered insufficient. In the current analysis, the KMO value was 0.856, indicating that a sufficiently large proportion of the variance is shared among the variables. In summary, both tests confirm the adequacy of PCA based on our data set.

Determination of the number of factors to be extracted

The number of factors to be extracted was determined according to the Kaiser-Guttman criterion, the Scree test, and parallel analysis [29].

The Kaiser-Guttman criterion is based on the eigenvalues of the factors. The eigenvalue of a factor is defined as the proportion of the variance of all observed standardized variables explained by that factor. It is obtained by summing the squared factor loadings across all variables. Since standardized variables have a variance of 1, an eigenvalue >1 means that this factor explains more variance than one variable alone. If this is the case, this factor can be used to reduce the data. The Kaiser criterion is based on this assumption, i.e., according to this criterion, all factors with an eigenvalue >1 are considered relevant. In our analysis, only the eigenvalue from the first factor was >1 (see Table S12.T1 below), which argues for the extraction of one factor.

| **Table S12.T1.** *Eigenvalues of all factors from exploratory principal component analysis* | | | | | | | | |
| --- | --- | --- | --- | --- | --- | --- | --- | --- |
| **Factor:** | 1 | 2 | 3 | 4 | 5 | 6 | 7 |  |
| **Eigenvalue:** | 3.573 | 0.898 | 0.696 | 0.602 | 0.504 | 0.374 | 0.353 |  |
| **Note.** The table shows the eigenvalues of each factor that could be extracted from the data of neuropsychological tests. | | | | | | | | |

The Scree Plot is generated by plotting the eigenvalue of each factor as a function of the number of that factor. All factors whose eigenvalues lie in front of the curve of the line in the scree plot are considered relevant.

**Figure S12.F1.** *Scree plot for data from neuropsychological tests.*


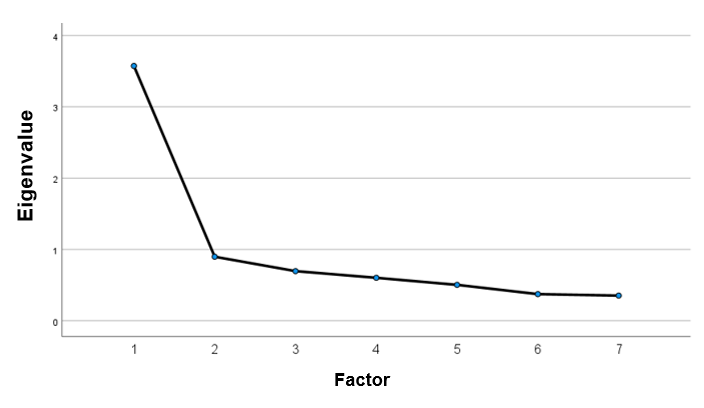


Parallel analysis (28) deals with the problem that eigenvalues >1 can be based on random correlations given in the data but are not observable in the population that these data are supposed to represent. To this end, the observed eigenvalues are compared with eigenvalues resulting from a parallel analysis with variables that are uncorrelated (orthogonal) in the population but have nonzero random correlations in the sample. We performed the parallel analysis using the SPSS macro rawpar [30]. The algorithm performs a 1000-fold permutation of the data used for factor analysis and extracts the eigenvalues of all possible factors at each iteration. Then, the eigenvalues found based on the actual data set are compared to the eigenvalues found based on the permuted data sets. The significance of the factors is calculated as the number of iterations in which the eigenvalue of a factor calculated based on the actual data set was smaller than the eigenvalues of the identical factor calculated based on the permuted data sets, divided by the number of iterations.

The parallel analysis (see Figure S12.F2) suggests the extraction of one factor. Since this result is consistent with the evaluation of the eigenvalues using the Kaiser criterion and the Scree plot, one factor was extracted as a result of the PCA.

**Figure S12.F2.** *Results from the parallel analysis.*


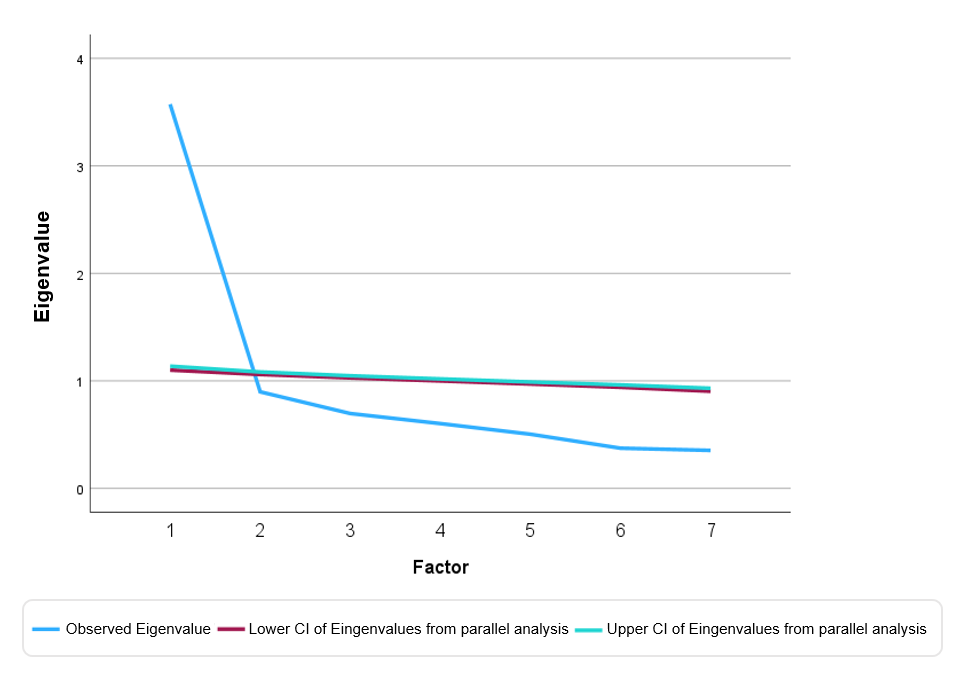


**Note.** The figure shows the results from the parallel analyses conducted to determine the number of factors to extract from the dataset. The blue line represents the eigenvalues calculated for each factor based on the actual dataset. The green and orange lines represent the upper and lower bound of the 95%-confidence interval (CI) of the eigenvalues calculated based on the permuted data sets.

## Extraction of factor scores

**Table S12.T2** *Factor loadings of the neuropsychological tests on the extracted factor.*

| **Test score** | **Primary cognitive domain** | **Factor loadings** |
| --- | --- | --- |
| DSST | Processing speed | 0.786 |
| TMT-A | Processing speed, visual scanning | 0.737 |
| D2 concentration | Selective attention | 0.762 |
| LNST | Verbal working memory | 0.628 |
| TMT-B | Cognitive flexibility, set-shifting | 0.786 |
| Corsi block fwd | Visuospatial memory span | 0.620 |
| Corsi block bw. | Visuospatial working memory | 0.620 |
| **Note.** DSST=Digit symbol substitution test, TMT=Trail making test, LNST=Letter number sequencing test, Corsi block fwd=Corsi block tapping test forward, Corsi block bw=Corsi block tapping test backwards. | | |

**Supplement 13: Regional distribution of the network**

**Figure S13.F1**. *Heatmaps of the regional distribution of brain regions.*

**
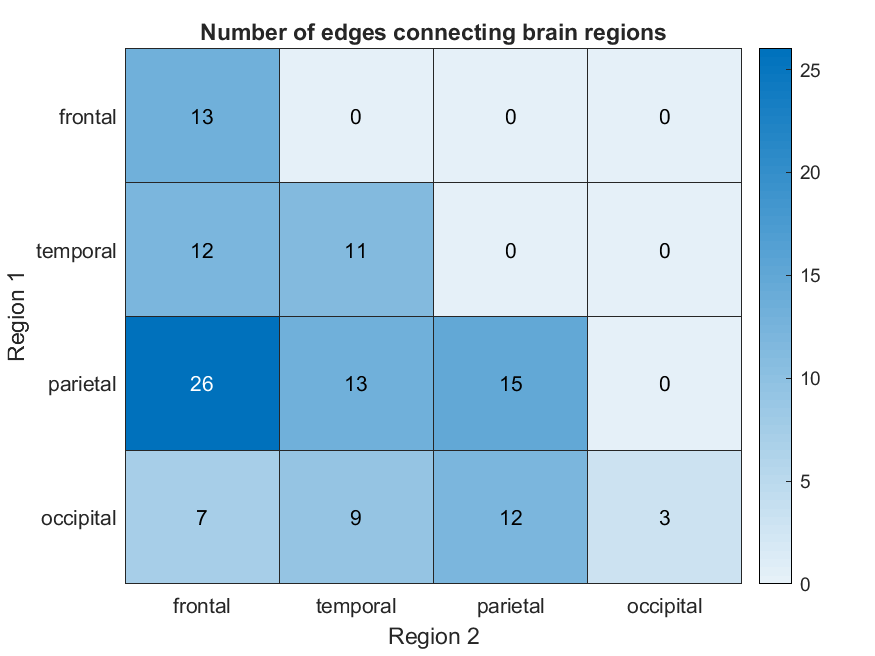
**

***Note.*** The figure shows the number of network edges from processing speed that connect frontal, temporal, parietal, and occipital brain regions.

**Table S13.T1**. *Network hubs.*

| **Node** | **Degree** |
| --- | --- |
| ctx-rh-parsorbitalis_1 | 9 |
| ctx-rh-inferiorparietal_3 | 7 |
| ctx-rh-superiorparietal_2 | 7 |
| ctx-rh-superiorparietal_3 | 7 |
| ctx-lh-parahippocampal_1 | 6 |
| ctx-lh-precuneus_2 | 6 |
| ctx-lh-superiorparietal_3 | 6 |
| ctx-rh-inferiortemporal_2 | 6 |
| ctx-rh-lateraloccipital_1 | 6 |
| ctx-lh-inferiorparietal_2 | 5 |
| ctx-rh-temporalpole_1 | 5 |
| ctx-lh-inferiortemporal_2 | 4 |
| ctx-lh-isthmuscingulate_1 | 4 |
| ctx-lh-lateraloccipital_1 | 4 |

***Note.*** The listed hubs are the top 15% of nodes with the highest degree within the processing speed network. Abbreviations: lh=light hemisphere; rh=right hemisphere.

**Supplement 14: Analyses based on different NBS *t*-thresholds**

**Figure S14.F1.** *Size of the processing speed-related subnetwork of white matter fiber tracts identified in the whole sample as a function of the primary threshold applied within the Network-based statistics toolbox.*

*
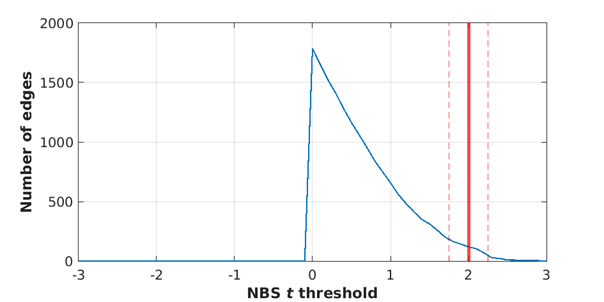
*

**Note.** The figure shows the size of the network identified with NBS when applying the respective *t*-threshold. NBS applies these thresholds to the test statistics representing the association between edge-wise connectivity strength (i.e., mean fractional anisotropy) and processing speed. If a given edge exceeds this threshold, it is included in the set of supra-threshold edges. The networks identified by NBS can only be composed of such supra-threshold edges. To evaluate the effect of the choice of threshold, we compared the effects reported in the article (i.e. NBS *t*-threshold=2.0) to those identified when applying the thresholds of *t*=1.75 and *t*=2.25.

| **Table S14.T1.** *Comparison of networks derived from Network-based statistic toolbox at different primary thresholds.* | | | |
| --- | --- | --- | --- |
|  | ***t*-threshold=1.75** | ***t*-threshold=2.0** | ***t-*threshold=2.25** |
| **Size of the network** | | | |
| Number of nodes | 100 | 89 | 54 |
| Number of edges | 176 | 121 | 58 |
| *p*_FWE_ | <.001 | <.001 | <.001 |
| **Association with processing speed** | | | |
| *t* value | 17.19 | 16.45 | 13.60 |
| *p* value | <.001 | <.001 | <.001 |
| partial *η²* | 0.175 | 0.163 | 0.117 |
| **Mediation analysis: Total effect of TNF-α PGS** | | | |
| coeff | -2.45 | -2.45 | -2.45 |
| 95%-CI | [-2.45, -0.77] | [-3.97, -0.93] | [-3.97, -0.93] |
| **Mediation analysis: Direct effect of TNF-α PGS** | | | |
| coeff | -1.79 | -1.65 | -1.91 |
| 95%-CI | [-3.17, -0.42] | [-3.05, -0.24] | [-3.36, -0.46] |
| **Mediation analysis: Indirect effect of TNF-α PGS** | | | |
| coeff | -0.66 | -0.80 | -0.54 |
| 95%-CI | [-1.30, -0.05] | [-1.45, -0.25] | [-1.11, -0.04] |
| **Note.** The table shows results from post-hoc analyses on the cognition-related subnetwork of white matter fiber tracts derived from the Network-based statistic toolbox when applying different primary thresholds. While the size of the network decreased when applying higher thresholds, the results of the association with processing speed in the whole sample as well as the mediation model with polygenic risk score for tumor necrosis factor-α (TNF-α PGS) as a predictor variable, structural brain connectivity as a mediator variable, and processing speed as an outcome variable in acute depressed patients remained largely unchanged. 95%-CI=95% confidence interval. | | | |

**Supplement 15: Robustness checks**

Robustness checks included correction for influential data points and clinical characteristics within patients. Furthermore, as an additional sensitivity check, we re-ran all analyses involving TNF-α PGS considering all eight ancestry components from the MDS analysis as covariates.

Correction for influential data points

To verify that our results were robust to influential data points, we repeated the analyses on the 1) positive association between mean FA and processing speed and 2) TNF-α PGS×diagnosis interaction effect on processing speed performance, but excluding participants with a Cook's distance >3*SD*. The analyses revealed that the pattern of results remained unchanged, even after the exclusion of 30 (*F*(1,1363)=316.03, *p*<.001, partial *η*²=.188) or 16 (*F*(3,1373)=5.56, *p*<.001, partial *η*²=.012) influential data points with a Cook's distance >3*SD.*

Correction for clinical characteristics within the patient sample

Several scores were used to adjust for typical clinical features associated with MDD: the number of previous hospitalizations reported during the interview to assess the cumulative illness severity, the Medication Load Index (MedIndex)[31] to assess the type and amount of current medication intake, as well as the presence of comorbid diagnoses (yes vs. no) assessed during the SCID-I interview. To this end, analyses were repeated, accounting for number of hospitalizations, MedIndex, and the presence of comorbid diagnoses in patients with MDD. Overall, the analyses showed that all effects maintained. More specifically, the analysis confirmed the significant positive association between mean FA and processing speed (*F*(1,639)=137.54, *p*<.001, partial *η*²=.177) as well as the significant TNF-α PGS×diagnosis interaction effect (*F*(2,636)=5.44, *p*=.005, partial *η*²=.017). Furthermore, repeating the mediation model in acute MDD patients confirmed the significant negative association between TNF-α PGS and processing speed (coeff=-2.70, 95%-CI [-4.18, -1.22], SE=0.75, *t*=-3.60, *p*<.001) and between TNF-α PGS and mean FA (coeff=-0.04, 95%-CI [-0.07, -.01], SE=0.02, *t*=-2.63, *p*=.009). Likewise, we observed a significant negative indirect (mediated) effect of TNF-α PGS on preprocessing speed through mean FA (coeff=-0.85, 95%-CI [-1.52, -0.25], SE=0.32). Finally, the model confirmed the significant direct effect of TNF-α PGS on preprocessing speed (coeff=-1.84, 95%-CI [-3.20, -0.49], SE=0.69).

Correction for eight MDS components

The analysis confirmed the significant TNF-α PGS×diagnosis interaction effect (*F*(3,1383)=4.14, *p*=.006, partial *η*²=.009), which was again driven by a negative association between TNF-α PGS and processing speed in the MDDa group (*B=*-2.42, *p*=.008, partial *η*²=.034; Bonferroni-corrected), whereas no association was found in the MDDpr (*p*=.348), MDDfr (*p*>.999), and HC group (*p*>.999). Furthermore, when repeating the mediation model in acute MDD, we again found a significant negative association between TNF-α PGS and processing speed (coeff=-2.42, 95%-CI [-3.96, -0.87], SE=0.78, *t*=-3.08, *p*=.002) and between TNF-α PGS and mean FA (coeff=-0.04, 95%-CI [-0.07, -.01], SE=0.02, *t*=-2.28, *p*=.022). Likewise, we observed a significant negative indirect (mediated) effect of TNF-α PGS on preprocessing speed through mean FA (coeff=-0.77, 95%-CI [-1.45, -0.19], SE=0.33). Finally, the model confirmed the significant direct effect of TNF-α PGS on preprocessing speed (coeff=-1.65, 95%-CI [-3.07, -0.24], SE=0.72). Overall, all effects remained significant even when accounting for all eight ancestry components

**Supplement 16: Exploratory analyses on sex-related differences**

In order to evaluate potential sex-related differences in the observed effects, all main analyses were repeated (1. differences in processing speed performance between diagnostic groups, 2. association between brain structural connectivity and processing speed performance, 3. association between TNF-α PGS and processing speed performance, and 4. mediator effect of brain structural connectivity on the association between TNF-α PGS and processing speed performance in acute MDD), including sex as a moderator variable to test potential differences in the effects between female and male participants. Results revealed no significant main effect of sex (*p*=.645) or a sex×diagnosis interaction (*p*=.418) on processing speed performance, indicating that the differences in processing speed performance do not vary between sexes. However, we found a significant main effect of sex on mean FA (*p*<.001, partial *η*²=.022), with female participants exhibiting lower mean FA compared to male participants. No sex×processing speed interaction on mean FA could be observed (*p*=.710), indicating that the association between processing speed performance and structural brain connectivity is comparable across females and males. We also did not find any significant main effect of sex (*p*=.910) or sex×TNF-α PGS interaction (*p*=.090), indicating that the association between TNF-α PGS and processing speed performance does not differ between female and male participants. Similarly, no sex×TNF-α PGS×diagnosis interaction effect was detected (*p*=.249). Lastly, a moderated mediation analysis was conducted in acute MDD, with sex as a moderator variable. The results did not show a moderated mediation effect by sex (95% CI [-0.15, 2.42]). Overall, the exploratory analyses yielded no indications for sex-related differences in the observed effects.

**Supplement 17: Cross validation of mediation model**

We have implemented a cross-site (Münster and Marburg) validation of our mediation model, which can provide an approximation of the generalizability. This approach has the advantage that all data were collected and processed with homogenized procedures and parameters. A failure of replication would therefore not be due to differences in data acquisition or processing, a well-known source of variance in neuroimaging research [32] and network neuroscience [33, 34].

For each site (Marburg vs. Münster) we, thus, calculated the processing speed factor as well as MDS components, and identified subnetworks of the structural connectome with edges associated with the site-specific processing speed factor. We then used the R package lavaan (Version 0.6-17; [35]) to estimate the full mediation model based on the Marburg data and used the estimated model to predict the processing speed factor value of each person from Münster. We then reversed this procedure by training a model in Münster and then predicting data from Marburg. In both cases, the accuracy of the prediction was evaluated using the root mean squared error (RMSE) and the correlation coefficient between the predicted and true values of the test set. To evaluate a given model’s accuracy, we implemented a permutation test in which the values of the processing speed factor in the training set were permuted before estimating and applying the model to the intact test data. This provided an estimate of the prediction accuracy of a model trained on random data in one site for intact test data from the other site. By repeating this procedure 1000 times per model, we obtained a null distribution of the accuracy based on random data, allowing us to determine *p*-values for the accuracies of that model.

Our analyses revealed that the mediation models trained separately in Marburg and Münster generalized well on the data from the other study site. For both models, we found lower than random deviation (Marburg-to-Münster: *RMSE*=0.978, *p*=0.016; Münster-to-Marburg: *RMSE*=1.009, *p*=0. 002) and a higher than random agreement (Marburg-to-Münster: *r*=0.373, *p*=0.034; Münster-to-Marburg: *r*=0.437, *p*=0.020) between the predicted and the observed processing speed values (see Figure below). We conclude that the significance of the mediation is not attributable to individual persons or study site-specific characteristics and, at least in the context of a bicentric, homogenized sample, also generalizes to unknown data. In our view, this in turn represents at least an approximation of the generalizability to other data sets.

**Figure S17.F1.** *Correlation between observed processing speed values and processing speed values predicted for Münster participants using a mediation model that was trained in Marburg.*


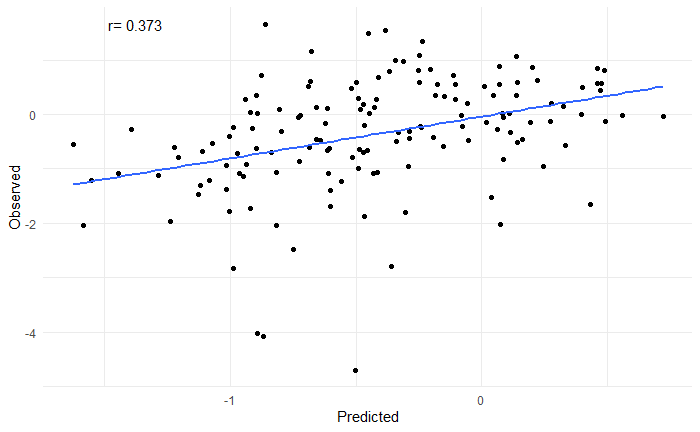


**Figure S17.F2.** *Correlation between observed processing speed values and processing speed values predicted for Marburg participants using a mediation model that was trained in Münster.*


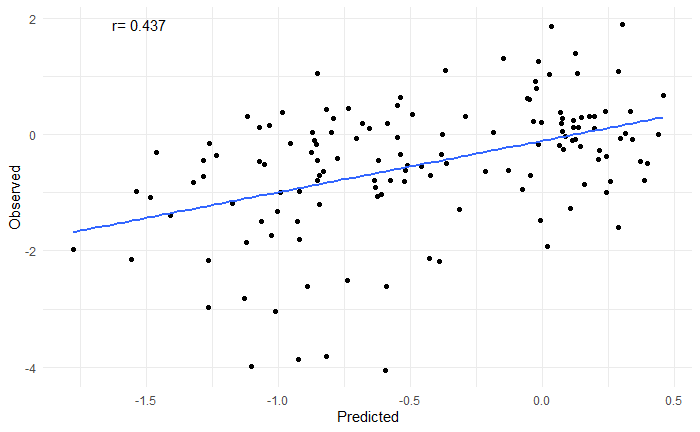


**Supplemental references**

1. Wittchen H-U, Wunderlich U, Gruschwitz S, Zaudig M. SKID I. Strukturiertes Klinisches Interview für DSM-IV. Achse I: Psychische Störungen. Interviewheft und Beurteilungsheft. Eine deutschsprachige, erweiterte Bearb. d. amerikanischen Originalversion des SKID I. 1997. 1997.

2. Harvey PD. Domains of cognition and their assessment. Dialogues Clin Neurosci. 2019;21:227–237.

3. Jaeger J. Digit Symbol Substitution Test. J Clin Psychopharmacol. 2018;38:513–519.

4. Wechsler D. Wechsler Adult Intelligence Scale--Fourth Edition. 2008. 2008. https://doi.org/10.1037/t15169-000.

5. Reitan R. Trail Making test: Manual for administration, scoring, and interpretation. Bloomingt Indiana Univ. 1956;Indianapolis: Indiana University Press.

6. Kessels RPC, van den Berg E, Ruis C, Brands AMA. The backward span of the Corsi Block-Tapping Task and its association with the WAIS-III Digit Span. Assessment. 2008;15:426–434.

7. Ross RM. The D2 Test of Attention: An Examination of Age, Gender, and Cross-cultural Indices. Argosy University; 2005.

8. Jenkinson M, Beckmann CF, Behrens TEJ, Woolrich MW, Smith SM. FSL. NeuroImage. 2012;62:782–790.

9. Smith SM, Jenkinson M, Woolrich MW, Beckmann CF, Behrens TEJ, Johansen-Berg H, et al. Advances in functional and structural MR image analysis and implementation as FSL. NeuroImage. 2004;23 Suppl 1:S208-219.

10. Woolrich MW, Jbabdi S, Patenaude B, Chappell M, Makni S, Behrens T, et al. Bayesian analysis of neuroimaging data in FSL. NeuroImage. 2009;45:S173-186.

11. Lange SC de, Heuvel MP van den. Structural and functional connectivity reconstruction with CATO - A Connectivity Analysis TOolbox. 2021:2021.05.31.446012.

12. Chang L-C, Jones DK, Pierpaoli C. RESTORE: robust estimation of tensors by outlier rejection. Magn Reson Med. 2005;53:1088–1095.

13. Chang L-C, Walker L, Pierpaoli C. Informed RESTORE: A method for robust estimation of diffusion tensor from low redundancy datasets in the presence of physiological noise artifacts. Magn Reson Med. 2012;68:1654–1663.

14. Heuvel MPVD, Sporns O, Collin G, Scheewe T, Mandl RCW, Cahn W, et al. Abnormal rich club organization and functional brain dynamics in schizophrenia. JAMA Psychiatry. 2013;70:783–792.

15. Gruber M, Mauritz M, Meinert S, Grotegerd D, de Lange SC, Grumbach P, et al. Cognitive performance and brain structural connectome alterations in major depressive disorder. Psychol Med. 2023:1–12.

16. Repple J, Gruber M, Mauritz M, Lange SC de, Winter NR, Opel N, et al. Shared and Specific Patterns of Structural Brain Connectivity Across Affective and Psychotic Disorders. Biol Psychiatry. 2022. 2022. https://doi.org/10.1016/j.biopsych.2022.05.031.

17. Cammoun L, Gigandet X, Meskaldji D, Thiran JP, Sporns O, Do KQ, et al. Mapping the human connectome at multiple scales with diffusion spectrum MRI. J Neurosci Methods. 2012;203:386–397.

18. Desikan RS, Ségonne F, Fischl B, Quinn BT, Dickerson BC, Blacker D, et al. An automated labeling system for subdividing the human cerebral cortex on MRI scans into gyral based regions of interest. NeuroImage. 2006;31:968–980.

19. Hagmann P, Cammoun L, Gigandet X, Meuli R, Honey CJ, Wedeen VJ, et al. Mapping the structural core of human cerebral cortex. PLoS Biol. 2008;6:e159.

20. Repple J, Mauritz M, Meinert S, de Lange SC, Grotegerd D, Opel N, et al. Severity of current depression and remission status are associated with structural connectome alterations in major depressive disorder. Mol Psychiatry. 2020;25:1550–1558.

21. Mori S, van Zijl PCM. Fiber tracking: principles and strategies - a technical review. NMR Biomed. 2002;15:468–480.

22. Sarwar T, Ramamohanarao K, Zalesky A. Mapping connectomes with diffusion MRI: deterministic or probabilistic tractography? Magn Reson Med. 2019;81:1368–1384.

23. de Reus MA, van den Heuvel MP. Estimating false positives and negatives in brain networks. NeuroImage. 2013;70:402–409.

24. Zalesky A, Fornito A, Cocchi L, Gollo LL, Heuvel MP van den, Breakspear M. Connectome sensitivity or specificity: which is more important? NEUROIMAGE. 2016;142:407–420.

25. van den Heuvel MP, Scholtens LH, van der Burgh HK, Agosta F, Alloza C, Arango C, et al. 10Kin1day: A Bottom-Up Neuroimaging Initiative. Front Neurol. 2019;10.

26. Zalesky A, Fornito A, Bullmore ET. Network-based statistic: identifying differences in brain networks. NeuroImage. 2010;53:1197–1207.

27. Freedman D, Lane D. A Nonstochastic Interpretation of Reported Significance Levels. J Bus Econ Stat. 1983;1:292–298.

28. Moosbrugger H, Schermelleh-Engel K. Exploratorische (EFA) und Konfirmatorische Faktorenanalyse (CFA). In: Moosbrugger H, Kelava A, editors. Testtheorie Fragebogenkonstruktion, Berlin, Heidelberg: Springer; 2012. p. 325–343.

29. Horn JL. A rationale and test for the number of factors in factor analysis. Psychometrika. 1965;30:179–185.

30. O’connor BP. SPSS and SAS programs for determining the number of components using parallel analysis and Velicer’s MAP test. Behav Res Methods Instrum Comput. 2000;32:396–402.

31. Redlich R, Almeida JR, Grotegerd D, Opel N, Kugel H, Heindel W, et al. Brain morphometric biomarkers distinguishing unipolar and bipolar depression. A voxel-based morphometry-pattern classification approach. JAMA Psychiatry. 2014;71:1222–1230.

32. Botvinik-Nezer R, Holzmeister F, Camerer CF, Dreber A, Huber J, Johannesson M, et al. Variability in the analysis of a single neuroimaging dataset by many teams. Nature. 2020;582:84–88.

33. Bryce NV, Flournoy JC, Guassi Moreira JF, Rosen ML, Sambook KA, Mair P, et al. Brain parcellation selection: An overlooked decision point with meaningful effects on individual differences in resting-state functional connectivity. NeuroImage. 2021;243:118487.

34. Gajwani M, Oldham S, Pang JC, Arnatkevičiūtė A, Tiego J, Bellgrove MA, et al. Can hubs of the human connectome be identified consistently with diffusion MRI? Netw Neurosci. 2023;7:1326–1350.

35. Rosseel Y. lavaan: An R Package for Structural Equation Modeling. J Stat Softw. 2012;48:1–36.
